# The Sequencing of Game Complexes in Women’s Volleyball

**DOI:** 10.3389/fpsyg.2020.00739

**Published:** 2020-04-30

**Authors:** Raúl Hileno, Marta Arasanz, Antonio García-de-Alcaraz

**Affiliations:** ^1^National Institute of Physical Education of Catalonia (INEFC), University of Lleida, Lleida, Spain; ^2^Faculty of Education Science, University of Almería, Almería, Spain; ^3^LFE Research Group, Universidad Politécnica de Madrid, Madrid, Spain

**Keywords:** match analysis, performance analysis, team sports, Markov chain, game sequence

## Abstract

In volleyball, each team must use no more than three hits to return the ball to the opponent’s court. This unique aspect of volleyball means that playing actions can be grouped into different complexes, mainly based on the initial defensive action. The purpose of this study was to find out which game complexes are most common in women’s volleyball and how those phases are sequenced. The study analyzed 4,252 complexes from 1,176 rallies or points (seven matches, with 27 sets in total) in the 2015 and 2016 Copa de la Reina. The variables analyzed were the game complex, complex efficacy, and number of complexes per point. Two Markov chains were defined to visualize how the complexes are sequenced. The first chain looked only at categories of the game complex variable, taking seven states and 24 transitions into consideration. The second chain combined the game complex and complex efficacy variables, taking 26 states and 125 transitions into consideration. These chains provide practical information regarding which sequences of complexes occur most frequently in the competition analyzed, and therefore which ones should be the main focus in training sessions. The most frequent sequence was Complex 0 (the serve), followed by Complex I with in-system attack, followed by Complex II without continuity.

## Introduction

Volleyball is a sport in which two teams compete against each other from opposite sides of a court divided by a net. The objective is to send the ball over the net and make it touch the floor in the opposite team’s court. Each team can use up to three hits (excluding the block and a consecutive contact at the first hit of a team) to send the ball over the net. The official rules published by the Fédération Internationale de Volleyball (2016) define a rally as “the sequence of playing actions from the moment of the service hit by the server until the ball is out of play” (p. 22). The playing actions that can take place during a rally are the serve, reception, set, attack, block, defense, and counterattack (Selinger and Ackermann-Blount, 1992). In both training and competition, these actions are usually grouped into game complexes (each of which is denoted by the letter K, followed by a numeral) so that the structure and dynamics of the game can be understood more easily (Rodríguez-Ruiz et al., 2011). Each type of complex differs from the others based primarily on the initial defensive action (Hileno and Buscà, 2012). Complex I (KI), for instance, starts with the service reception and continues with the set and the attack. Complex II (KII), on the other hand, starts with the attack defense and continues with a set and a counterattack.

Initially, when the old *side-out scoring system* was used in the twentieth century, coaches only distinguished between two types of complexes when analyzing matches and organizing training sessions (Zhang, 2000). The first was the *side-out phase* (KI), which was performed by the team that was receiving serve, with the aim of recovering serve and rotating. The second was the *point phase* (KII), which was performed by the team that was serving, with the aim of scoring a point and continuing to serve (Valladares et al., 2016). In the twenty-first century, following the introduction of the *rally point scoring system* in 1998, research on volleyball (Hurst et al., 2016, 2017; Laporta et al., 2018a, b) and beach volleyball (Medeiros et al., 2017) has identified six game complexes. These complexes can also be used for planning and organizing technical and tactical training. The four new complexes, in addition to KI and KII, are Complex 0 (K0), which is also known as the serve, and complexes III, IV, and V (KIII, KIV, and KV), which begin, respectively, with a counterattack defense, an offensive block defense, and a free-ball defense, and continue with a set and then a counterattack (Hileno and Buscà, 2012).

Specialized theoretical literature on volleyball that diagrams the sequencing of game actions (e. g., Eom and Schutz, 1992; Selinger and Ackermann-Blount, 1992; Meier, 1994; Marcelino et al., 2008) is more plentiful than the literature showing the sequencing of game complexes. Palao et al. (2004) produced a flowchart with only three game complexes (KI, KII, and KIII). Laporta et al. (2018a) developed another flowchart with six game complexes (labeled from K0 to KV), from which the authors identified a total of 13 transitions between complexes (K0 to KI; KI to KII, KIV, and KV; KII to KIII and KIV; KIII to KIII, KIV, and KV; KIV to KIII and KV; and KV to KIII and KIV). Unfortunately, the diagrams did not show which complex transitions were the most frequent and which needed to be worked on the most in training.

Various researchers recently addressed this shortcoming by using social network diagrams and measuring eigenvector centrality to analyze the connections between game complexes in elite women’s and men’s volleyball (Hurst et al., 2016, 2017; Loureiro et al., 2017; Laporta et al., 2018a, b). In addition to the game complex variable, the researchers considered other variables that are established during a rally, such as the serve type, serve zone, reception zone, setting zone, and number of attackers available. One key variable, however, is absent from their match analyses: complex efficacy. Lames and McGarry (2007) analyzed the transitions between six game actions (serve, reception, set, attack, block, and dig), taking into account their efficacy (winning points) and analyzing interactions using a Markov chain. This approach is similar to social networks, and is based on the study of transition probabilities between events or states (Isaacson and Madsen, 1976). Therefore, the purpose of the present study was to find out which game complexes are most frequent in women’s volleyball and how those complexes are sequenced, analyzing their efficacy and transitions using Markov chains.

## Materials and Methods

### Participants

We analyzed 4,252 game complexes from 1,176 points in two matches (six sets) in the 2015 Copa de la Reina (the Spanish national knockout cup competition in women’s volleyball) and five matches (21 sets) in the 2016 competition. The matches involved seven teams: CVB Barça, Feel Volley Alcobendas, Fígaro Peluqueros Haris, GH Leadernet Navarcable, Haro Rioja Voley, Naturhouse Ciudad de Logroño, and VP Madrid. All teams used a 5-1 offensive formation, a two- or three-player serve-receive pattern, and a player-back defensive system (USA Volleyball, 2009). Each rally began with the serve and ended when the first referee indicated that the ball was out of play. Complex 0 was recorded as soon as a player served the ball and the other complexes were recorded straight after the first touch of each possession, unless the point ended earlier as per the rules of the game.

### Variables

The *game complex* refers to the period during which one team is in possession of the ball (Laporta et al., 2015). Following Hurst et al. (2016, 2017), Loureiro et al. (2017), and Laporta et al. (2018a, b), this study distinguished between six game complexes: (a) Complex 0 (K0: serve); (b) Complex I (KI: service reception, set, and attack); (c) Complex II (KII: attack defense, set, and counterattack); (d) Complex III (KIII: counterattack defense, set, and counterattack); (e) Complex IV (KIV: offensive block defense, set, and counterattack); and (f) Complex V (KV: free-ball defense, set, and counterattack). The present study adds a seventh complex, called “undefined complex” (UK), to capture complexes that were hard to classify, since they began with an unusual defensive action, such as defending an “overpass spike” or a “joust.” An overpass spike occurs when a player spikes an overpass that is just above the net due to a poor reception, defense, or set by the opposite team; a joust occurs when at least one player from each team simultaneously makes contact with a ball that is just above the net after a poor first or second hit.

The *complex efficacy* indicates how well the observed game complex is executed. This study uses an adaptation of the ordinal scale proposed by Palao et al. (2015), with four categories to evaluate the efficacy of the game complex: (a) no continuity (0: the complex does not allow play to continue); (b) no spike (1: the complex does not allow the attack or counterattack to culminate with a spike, ending instead with a free-ball); (c) out-of-system offense (2: the complex allows the attack or counterattack to culminate with a spike, but the build-up to the spike is disorganized because the set is performed by a player other than the setter or because the setter receives a poor pass, in other words, a pass that goes to the setter’s forearms, a pass that goes to a position that is not ideal for setting, or a pass that leaves the middle hitter unavailable); and (d) in-system offense (3: the complex allows the attack or counterattack to culminate with a spike following a well-organized build-up, with the first hit being good enough to allow the setter to perform a dump on the second touch or to deliver a good-quality set from an ideal court position, with the middle hitter available).

The variable for the *number of complexes per point* indicates the total number of complexes between the ball being served and the first referee indicating that the ball is out of play. If the server did not produce a legal serve, only Complex 0 was recorded. This meant that at least one game complex was recorded for each point.

### Procedures

The two competitions were recorded using a digital video camera on a tripod located in the center of one of the stands situated at one end of the court. The recorded video files were played back using the sports video analysis software Kinovea v. 0.8.15 (Joan Chartman and contributors, Free Software Foundation, Inc., Boston, MA, United States) and the data were recorded in a Microsoft Excel 2013 (Microsoft Corp., Redmond, WA, United States) spreadsheet.

To analyze the reliability of the data, a set from one match in the sample was selected at random. The chosen set had 154 game complexes and 41 points. Two observers – both certified volleyball coaches – recorded data for the set. The first observer recorded the data, then did the same again 2 weeks later; the second observer recorded data only once. Before this reliability test, both observers trained for 2 h to familiarize themselves with the log sheet and the variables being studied. Agreement among the observations was estimated using Cohen’s kappa coefficient. The coefficient was greater than 0.97 for all three variables both in the comparison of the two observations by observer 1 and in the comparison between observers 1 and 2. In accordance with the scale proposed by Landis and Koch (1977), the strength of agreement was therefore *almost perfect* for all three variables analyzed.

### Statistical Analysis

Firstly, a descriptive analysis was carried out for two variables: number of complexes per point and game complex. For the game complex variable, the confidence interval for a proportion (1-α confidence interval for π) was also calculated using the Wilson method.

Secondly, the relationship between the game complex and complex efficacy variables (and their respective categories) was studied using Pearson’s chi-squared test (χ^2^), the corrected contingency coefficient (*C*_corr_), and adjusted residuals or Allison and Liker *z* scores (Allison and Liker, 1982). The corrected contingency coefficient was used to determine whether the strength of the relationship was weak (*C*_corr_ < 0.30), moderate (*C*_corr_ ≥ 0.30 and ≤ 0.70), or strong (*C*_corr_ > 0.70) (Kraska-Miller, 2014).

Finally, two Markov chains or state transition diagrams were constructed to show transition probabilities (*p*_*ij*_) between different game complexes. The first chain took complex efficacy into account; the second did not. According to Isaacson and Madsen (1976), a Markov chain is a discrete stochastic process in which the probability of an event occurring (state *j*) depends only on the immediately preceding event (state *i*). In mathematical terms, the probability of moving from state *i* to state *j* is expressed as follows:

$$p_{\mathrm{ij}}=\mathrm{Pr}\left(x_{1}=j|x_{0}=i\right)$$

These transition probabilities (*p*_ij_) were calculated from two two-dimensional contingency tables, applying the following equation in each table cell:

$$p_{\mathrm{ij}}=n_{\mathrm{ij}}/n_{i}$$

where *n*_ij_ is the frequency observed in the cell located in row *i* and column *j* in the contingency table, and *n*_xi_ is the total frequency of row *i* in the contingency table.

All the statistical analysis was performed in Stata/IC v. 15.1 (StataCorp, College Station, TX, United States) and the Markov chains were performed in MATLAB v. 9.6 (The MathWorks, Inc., Natick, MA, United States). A significance level of 0.05 was used for all the statistical tests.

## Results

The mean number of complexes per point was 3.62, with a standard deviation of 1.78. Every point had from 1 to 13 complexes. The median number of complexes was 3 and the interquartile interval was 3–4.

The most frequent numbers of complexes per point were 3 (45.1%), 4 (16.5%), 5 (10.7%), 1 (9.5%), 2 (6.8%), and 6 (5.2%). Only 6.2% of the points had from 7 to 13 complexes.

As shown in Table 1, K0 was the most frequent game complex (27.66%), followed by complexes KI, KII, KIII, KIV, KV, and UK.

**TABLE 1:** A descriptive analysis (absolute and relative frequencies) and inferential analysis (confidence interval for a proportion calculated using the Wilson method) of the game complex variable.

			95% CI	
Game complex	*n*	%	*LL*	*UL*
Complex 0	1,176	27.66	26.33	29.02
Complex I	1,064	25.02	23.74	26.35
Complex II	711	16.72	15.63	17.87
Complex III	680	15.99	14.92	17.12
Complex IV	280	6.59	5.88	7.37
Complex V	249	5.86	5.19	6.60
Undefined	92	2.16	1.77	2.65

**n*, number of observations; CI, confidence interval; *LL*, lower limit; *UL*, upper limit.*

A significant moderate relationship was found between the game complex and complex efficacy variables (χ^2^ = 1,093.49, *df* = 15, *p* < 0.001, *C*_corr_ = 0.59). Furthermore, eight significant positive relationships were found between categories of these two variables (adjusted residuals or *z* scores ≥1.96). As shown in Table 2, the end of a point (no continuity) was associated with KII, KIII, KIV, and UK; a free-ball (no spike) at the end of a complex was associated with KIV; a disorganized counterattack (out-of-system offense) at the end of a complex was associated with KIII; and an attack or counterattack (in-system offense) at the end of a complex was associated with KI and KV, respectively.

**TABLE 2:** Relationships between the game complex and complex efficacy variables (adjusted residuals).

	Game complex					
Complex performance	Complex I	Complex II	Complex III	Complex IV	Complex V	Undefined
No continuity	−22.95***	15.38***	10.03***	6.34***	−8.77***	4.94***
No spike	–0.52	–0.14	–0.36	2.96**	–1.83	0.60
Out-of-system offense	1.07	–1.34	2.52*	–1.55	–1.05	–1.52
In-system offense	23.39***	−14.74***	−12.68***	−7.22***	11.46***	−4.13***

*p < 0.05, **p < 0.01, ***p < 0.001.

**FIGURE 1:**
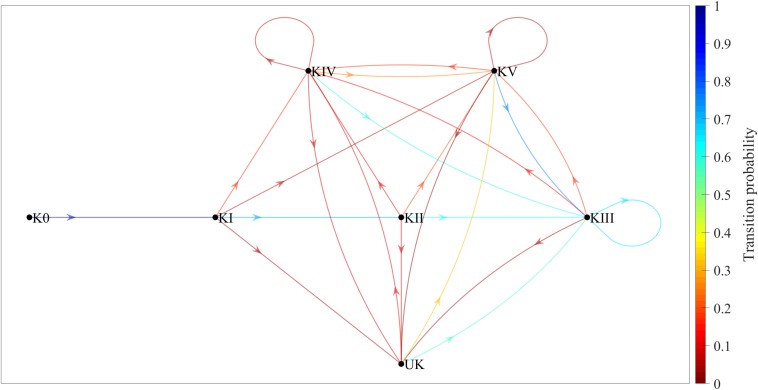
Game-complex transition probabilities (without taking complex efficacy into account) shown in a Markov chain consisting of seven states and 24 transitions. K0, complex 0; KI, complex I; KII, complex II; KIII, complex III; KIV, complex IV; KV, complex V; UK, undefined complex.

Figure 1 shows a Markov chain for the game complex variable only, with seven states and 24 transitions. The colors show the transition probabilities between different states, as per the color scale on the right. The conditional probability, for example, of KII occurring after KI has occurred is about 0.70. In other words, *p*(KII | KI) ≈ 0.70.

**FIGURE 2:**
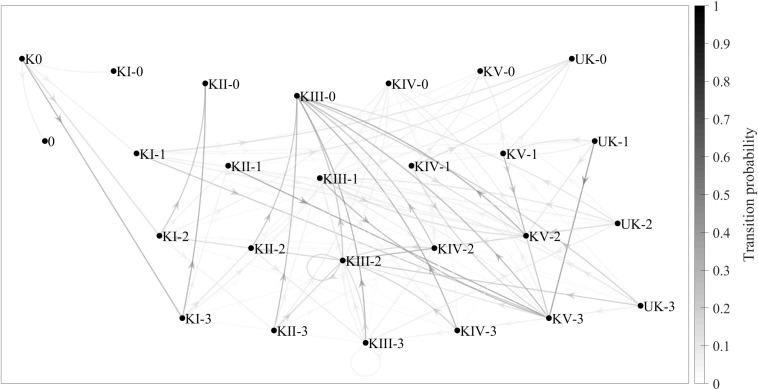
Game-complex transition probabilities (taking complex efficacy into account) shown in a Markov chain consisting of 26 states and 125 transitions. 0, no continuity; 1, no spike; 2, out-of-system offense; 3, in-system offense.

Figure 2 shows another Markov chain, this time combining the game complex and complex efficacy variables, with 26 states and 125 transitions. The grayscale tones show the transition probabilities between different states, as per the grayscale bar on the right. The conditional probability, for example, of KII without continuity occurring after KI with in-system attack has occurred is about 0.50. In other words, *p*(KII-0 | KI-3) ≈ 0.50.

## Discussion

The main objective of this study was to understand how game complexes are sequenced in women’s volleyball by analyzing them using Markov chains – a different approach from social networks. Although the structure might look identical, the information that a Markov chain (state transition diagram) provides is not exactly the same as what a social network (sociogram) provides. Both processes involve a series of dots, points, events, states, nodes, or vertices connected to each other by lines, arrows, transitions, edges, tiles, bridges, links, or connections. In sociograms, however, the size of the nodes tends to be modified to indicate the node degree (number of edges connected to a node) and the thickness of the edges tends to be modified to indicate the weight or “cost” of the connections in the network (Newman, 2003). In state transition diagrams, on the other hand, the size of the states and the thickness of the transitions are normally unmodified, with transition probabilities between adjacent states indicated using numbers or colors.

Regarding the number of game complexes per point, almost half of the points in the present study had only three game complexes each. This fact and the transition probabilities shown in the first Markov chain (Figure 1) show that the most frequent sequence of complexes is K0→KI→KII, followed by the sequences K0→KI→KIV, K0→KI→KV, and K0→KI→UK. Consequently, for training to reflect competition play as closely as possible, KI training sessions should work on approximately seven K0→KI→KII sequences, two K0→KI→KIV sequences, and one K0→KI→KV or K0→KI→UK sequence for every ten serves.

João et al. (2010) indicate that the mean length of points is usually longer in women’s volleyball than in the men’s game due to the different anthropometric and physiological characteristics of men and women. Indeed, in the present study, long rallies with seven or more complexes were rare, but long rallies with four to six complexes were fairly common, since they accounted for a third of all points. In practical terms, this finding suggests that ball control drills using a net should not require women players to practice long rallies with more than six complexes or ball exchanges over the net.

Laporta et al. (2018a) found that the most frequent game complex in elite women’s volleyball was K0, followed in order of frequency by KI, KII, KIII, KV, and KIV. In the present study, however, the order of frequency was K0, KI, KII, KIII, KIV, KV, and UK. One possible reason why KIV was more frequent than KV in this study, rather than the other way around, is the addition of the undefined complex (UK). In other words, if the present study had not defined a separate UK category and such complexes had been categorized as KV (which to a certain extent would make sense, since both are the result of a poor first or second hit in the previous complex), KV would also have been more frequent than KIV, as was the case in the aforementioned study. Another possible explanation might be the performance level of players analyzed, who are non-elite, in contrast to the elite players observed by Laporta et al. (2018a).

The analysis of the relationship between game complexes and their efficacy found that KI and KV were most closely associated with an in-system offense, whereas KII, KIII, KIV, and UK were most closely associated with no continuity. This may be because the defensive actions that initiate KI (receiving a serve) and KV (defending a free-ball) start further away from the net, thus allowing a longer reaction time than the defensive actions that initiate KII (defending an attack), KIII (defending a counterattack), KIV (defending an offensive block), and UK (defending an overpass spike or a joust). Castro and Mesquita (2010) argue that the further away the opposite team’s offensive action is from the net, the easier it is to produce a better-quality first touch and then to build a well-organized attack or counterattack.

The Markov chain in Figure 1 is less complex and easier to interpret than the one in Figure 2, since there are fewer transitions between complexes (24 in Figure 1 vs. 125 in Figure 2). The first chain, however, is less accurate than the second one, since it only covers transient (K0, KI, and KII) and recurrent (KIII, KIV, KV, and UK) states without ever completing the sequence initiated with K0. Despite its complexity, the second chain is a much better reflection of the reality of the game, since in addition to transient and recurrent states, it also includes absorbing states such as KI-0 (Complex I without continuity), in which the sequence begins with a serve and ends with an error made during KI.

There are two other important matters to consider regarding the first Markov chain, which did not take complex efficacy into account: (a) there were seven main transitions between complexes (K0 to KI; KI to KII; and KII, KIII, KIV, KV, and UK to KIII), each with a transition probability of around 0.60 or higher; and (b) even without taking into account the transitions involving the UK, this Markov chain showed three transitions (KII to KV, KIV to KIV, and KV to KV) that were not present in the theoretical diagram by Laporta et al. (2018a). Furthermore, in the second Markov chain, which did take complex efficacy into account, there are two additional aspects that should be considered: (a) the most relevant sequence of complexes was K0→KI-3→KII-0 (K0 [the serve], followed by KI with in-system attack, followed by KII without continuity), because the transition probability between the states was high (close to 0.50) and because the most common number of complexes per point in the present study was three; and (b) the most likely transition other than KII-3, KIII-3, KIV-3, KV-3, and UK-3 (that is, KII, KIII, KIV, KV, and UK, each with in-system counterattack) was KIII-0 (Complex III without continuity), which means that a good counterattack is more likely than not to result in the point ending during the next complex.

Finally, despite the contributions of the present study, a number of methodological constraints have been identified that could be covered in future research. First, only senior women’s volleyball at the national level was analyzed. Future studies should therefore check whether game complexes follow similar sequences in men’s volleyball, in other age categories, and at other levels of competition (initial stages and top-levels). Second, the transitions between complexes were analyzed without taking into account contextual variables such as quality of opposition, match status, and match period, even though performance analysis in team sports such as soccer (García-Unanue et al., 2018), basketball (Sampaio et al., 2010), and volleyball (Marcelino et al., 2012) does take them into account.

## Conclusion

The research presented in this paper found that the most frequent number of game complexes per point in the national championship analyzed is three. The most common complex is K0, followed by KI, KII, KIII, KIV, KV, and UK, in that order. KI and KV are associated with an in-system offense, while KII, KIII, KIV, and UK are more closely associated with no continuity. The most common sequence of complexes is K0 (the serve), followed by KI with in-system attack, followed by KII without continuity.

The methodology of analyzing game-complex transitions using Markov chains is considered a valid alternative to the social networks methodology, providing practical information about which sequences are most likely to occur in competition, thus indicating which sequences training sessions should focus on the most. The two Markov chains presented in this paper show that the sequencing of game complexes in competition is not as straightforward as various theoretical diagrams have traditionally proposed. Moreover, the Markov chain that takes complex efficacy into account reflects the reality of the game of volleyball much better than the one that does not.

## Practical Implications

The main contributions of this paper that coaches can apply in training are as follows: (a) the game complexes that are most important for the analyzed women’s volleyball teams to work on are K0 and KI, followed by KII and KIII; (b) KIV should be worked on slightly more than KV; (c) teams looking to work on short rallies should focus in particular on the K0→KI-3→KII-0 sequence, with a controlled serve to make it easier for the team receiving serve to build an in-system offense; and (d) teams looking to work on long rallies should carry out ball control drills in which the ball crosses the net four to six times in total, with the sequence starting with a poor reception of serve or a poor defense, resulting in a free-ball or an out-of-system offense. The findings would allow designing an adequate training session following specific competition demands. Moreover, trainers or strength and condition coaches may find in this study a useful guide of the most frequent movements and the sequence of actions demanded in these game complexes. Thus, the development of youth players and/or the sign up of new players may develop according to the demands of this specific context.

## Data Availability Statement

The datasets generated for this study are available on request to the corresponding author.

## Ethics Statement

Ethical review and approval was not required for the study on human participants in accordance with the local legislation and institutional requirements. Written informed consent from the participants was not required to participate in this study in accordance with the national legislation and the institutional requirements.

## Author Contributions

RH worked on the conceptualization and design of the study, analysis and interpretation of the data, and drafting of the manuscript. MA worked on the collection and interpretation of the data, and drafting of the manuscript. AG participated in the data interpretation and reviewed the content of the manuscript.

## Conflict of Interest

The authors declare that the research was conducted in the absence of any commercial or financial relationships that could be construed as a potential conflict of interest.

## References

[B1] Allison P. D. Liker J. K. (1982). Analyzing sequential categorical data on dyadic interaction: a comment on Gottman. *Psychol. Bull.* 91 393–403. 10.1037/0033-2909.91.2.393

[B2] Castro J. M. Mesquita I. (2010). Analysis of the attack tempo determinants in volleyball’s complex II - A study on elite male teams. *Int. J. Perf. Anal. Spor.* 10 197–206. 10.1080/24748668.2010.11868515

[B3] Eom H. J. Schutz R. W. (1992). Statistical analyses of volleyball team performance. *Res. Q. Exercise Sport.* 63 11–18. 10.1080/02701367.1992.10607551 1574656

[B4] Fédération Internationale de Volleyball. (2016). *Official Volleyball Rules 2017-2020: Approved by the 35th FIVB Congress 2016.* Available online at: https://www.fivb.com/en/volleyball/thegame_glossary/officialrulesofthegames

[B5] García-Unanue J. Pérez-Gómez J. Giménez J. V. Felipe J. L. Gómez-Pomares S. Gallardo L. (2018). Influence of contextual variables and the pressure to keep category on physical match performance in soccer players. *PLoS One.* 13:e0204256. 10.1371/journal.pone.0204256 30235298 PMC6147482

[B6] Hileno R. Buscà B. (2012). Observational tool for analyzing attack coverage in volleyball. *Rev. Int. Med. Cienc. AC*. 12 557–570. Available online at: http://cdeporte.rediris.es/revista/revista60/artRIMCAFD639.htm

[B7] Hurst M. Loureiro M. Valongo B. Laporta L. Nikolaidis P. T. Afonso J. (2016). Systematic mapping of high-level women’s volleyball using social network analysis: the case of serve (K0), side-out (KI), side-out transition (KII) and transition (KIII). *Int. J. Perf. Anal. Spor.* 16 695–710. 10.1080/24748668.2016.11868917

[B8] Hurst M. Loureiro M. Valongo B. Laporta L. Nikolaidis P. T. Afonso J. (2017). Systematic mapping of high-level women’s volleyball using social network analysis: the case of attack coverage, freeball, and downball. *Monten. J. Sports Sci. Med.* 6 57–64.

[B9] Isaacson D. L. Madsen R. W. (1976). *Markov Chains: Theory and Applications.* New York, NY: Wiley.

[B10] João P. V. Leite N. Mesquita I. Sampaio J. (2010). Sex differences in discriminative power of volleyball game-related statistics. *Percept. Mot. Skills* 111 893–900. 10.2466/05.11.25.PMS.111.6.893-900 21319626

[B11] Kraska-Miller M. (2014). *Nonparametric Statistics for Social and Behavioral Sciences.* Boca Raton, FL: CRC Press.

[B12] Lames M. McGarry T. (2007). On the search for reliable performance indicators in game sports. *Int. J. Perf. Anal. Spor.* 7 62–79. 10.1080/24748668.2007.11868388

[B13] Landis J. R. Koch G. G. (1977). The measurement of observer agreement for categorical data. *Biometrics* 33 159–174. 10.2307/2529310 843571

[B14] Laporta L. Afonso J. Mesquita I. (2018a). Interaction network analysis of six game complex in high-level volleyball through the use of eigenvector centrality. *PLoS One.* 13:e0203348. 10.1371/journal.pone.0203348 30204789 PMC6133287

[B15] Laporta L. Afonso J. Mesquita I. (2018b). The need for weighting indirect connections between game variables: social network analysis and eigenvector centrality applied to high-level men’s volleyball. *Int. J. Perf. Anal. Spor.* 18 1067–1077. 10.1080/24748668.2018.1553094

[B16] Laporta L. Nikolaidis P. Thomas L. Afonso J. (2015). Attack coverage in high-level men’s volleyball: organization on the edge of chaos? *J. Hum. Kinet.* 47 249–257. 10.1515/hukin-2015-0080 26557208 PMC4633260

[B17] Loureiro M. Hurst M. Valongo B. Nikolaidis P. Laporta L. Afonso J. (2017). A comprehensive mapping of high-level men’s volleyball gameplay through social network analysis: analysing serve, side-out, side-out transition and transition. *Monten. J. Sports Sci. Med.* 6 35–41. 10.26773/mjssm.2017.09.005

[B18] Marcelino R. Mesquita I. Sampaio J. (2008). Estudo dos indicadores de rendimento em voleibol masculino em função do número do set. *R. Bras. Ci. e Mov.* 16 1–23. 10.18511/rbcm.v16i3.841

[B19] Marcelino R. Sampaio J. Mesquita I. (2012). Attack and serve performances according to the match period and quality of opposition in elite volleyball matches. *J. Strength Cond. Res.* 26 3385–3391. 10.1519/JSC.0b013e3182474269 22207260

[B20] Medeiros A. I. A. Marcelino R. Mesquita I. M. Palao J. M. (2017). Performance differences between winning and losing under-19, under-21 and senior teams in men’s beach volleyball. *Int. J. Perf. Anal. Spor.* 17 96–108. 10.1080/24748668.2017.1304029

[B21] Meier M. (1994). Movement dynamics in volleyball with young players. *Int. Volley Tech.* 1 11–16. 30317919

[B22] Newman M. E. J. (2003). The structure and function of complex networks. *SIAM Rev. Soc. Ind. Appl. Math.* 45 167–256. 10.1137/S003614450342480

[B23] Palao J. M. Manzanares P. Ortega E. (2015). Design and validation of an observation instrument for technical and tactical actions in indoor volleyball. *Eur. J. Hum. Mov.* 34 75–95. 30733691

[B24] Palao J. M. Santos J. A. Ureña A. (2004). Effect of team level on skill performance in volleyball. *Int. J. Perf. Anal. Spor.* 4 50–60. 10.1080/24748668.2004.11868304

[B25] Rodríguez-Ruiz D. Quiroga M. E. Miralles J. A. Sarmiento S. Saá de Y. García-Manso J. M. (2011). Study of the technical and tactical variables determining set win or loss in top-level European men’s volleyball. *J. Quant. Anal. Sports* 7 1–13. 10.2202/1559-0410.1281

[B26] Sampaio J. Lago C. Casais L. Leite N. (2010). Effects of starting score-line, game location, and quality of opposition in basketball quarter score. *Eur. J. Sport Sci.* 10 391–396. 10.1080/17461391003699104

[B27] Selinger A. Ackermann-Blount J. (1992). *Power Volleyball.* Paris: Vigot.

[B28] USA Volleyball. (2009). *Volleyball Systems & Strategies.* Champaign, IL: Human Kinetics.

[B29] Valladares N. García-Tormo J. V. João P. V. (2016). Analysis of variables affecting performance in senior female volleyball world championship 2014. *Int. J. Perf. Anal. Spor.* 16 400–410. 10.1080/24748668.2016.11868895

[B30] Zhang R. (2000). How to profit by the new rules. *The Coach.* 1 9–11.

